# Faster R-CNN for Robust Pedestrian Detection Using Semantic Segmentation Network

**DOI:** 10.3389/fnbot.2018.00064

**Published:** 2018-10-05

**Authors:** Tianrui Liu, Tania Stathaki

**Affiliations:** Department of Electrical and Electronic Engineering, Imperial College London, London, United Kingdom

**Keywords:** pedestrian detection, deep learning, convolutional neural network, semantic segmentation, region proposal

## Abstract

Convolutional neural networks (CNN) have enabled significant improvements in pedestrian detection owing to the strong representation ability of the CNN features. However, it is generally difficult to reduce false positives on hard negative samples such as tree leaves, traffic lights, poles, etc. Some of these hard negatives can be removed by making use of high level semantic vision cues. In this paper, we propose a region-based CNN method which makes use of semantic cues for better pedestrian detection. Our method extends the Faster R-CNN detection framework by adding a branch of network for semantic image segmentation. The semantic network aims to compute complementary higher level semantic features to be integrated with the convolutional features. We make use of multi-resolution feature maps extracted from different network layers in order to ensure good detection accuracy for pedestrians at different scales. Boosted forest is used for training the integrated features in a cascaded manner for hard negatives mining. Experiments on the Caltech pedestrian dataset show improvements on detection accuracy with the semantic network. With the deep VGG16 model, our pedestrian detection method achieves robust detection performance on the Caltech dataset.

## 1. Introduction

Object detection is a fundamental problem in computer vision and has wide applications in video surveillance (Jian et al., 2013; Jian and Lam, 2015), robotics automation, and intelligence transportation. In particular, pedestrian detection is of great interest to both research and industry owing to its practical applications to driver assistance systems and intelligent video surveillance. For video surveillance, pedestrian detection helps to provide fundamental information for people counting, event recognition, and crowd monitoring; for intelligent transportation, pedestrian detection is an essential part for the semantic understanding of the environment.

**Figure 1:**
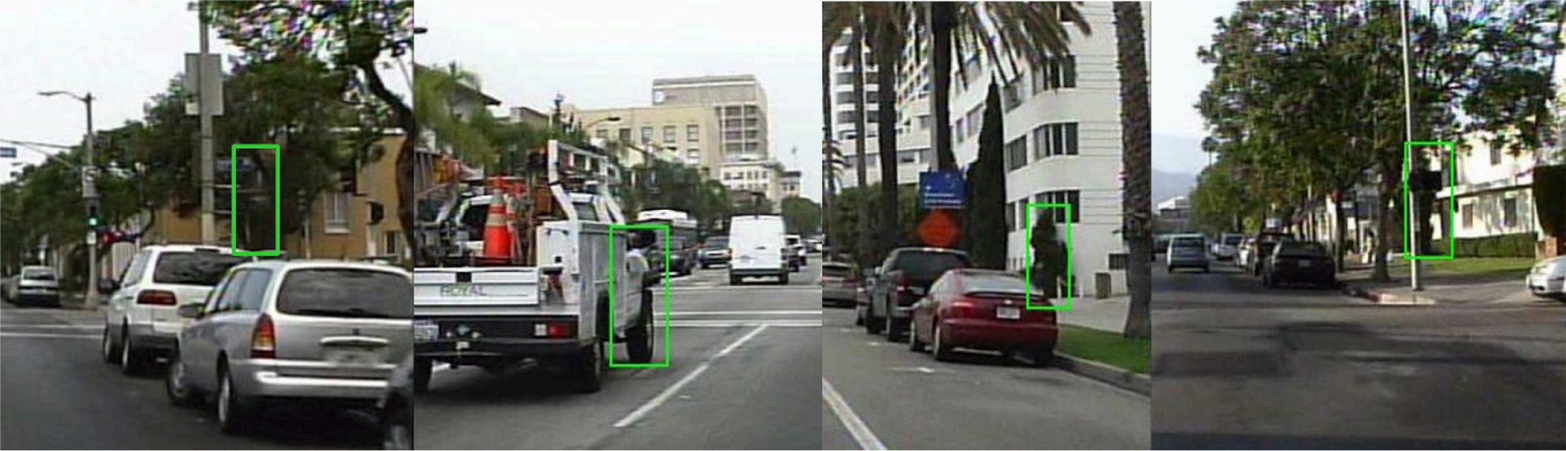
Examples of false positives on tree leaves and vehicle from the Caltech dataset (Dollár et al., 2009b).

Pedestrian detection is often challenged by significant intra-class variability since human tend to have greatly variations in human pose and appearance. A substantial number of methods have been developed in order to improve detection accuracy (Dalal and Triggs, 2005; Felzenszwalb et al., 2010; Dollár et al., 2014; Li et al., 2015; Zhang et al., 2015, 2016b,a; Costea and Nedevschi, 2016; Liu and Stathaki, 2016, 2017). However, pedestrian detectors still suffer from a relatively high rate of hard negatives such as tree leaves, traffic lights when solely using pedestrian features (see examples in Figure 1). Some of these false negatives can be removed by making use of higher level vision cues, i.e., semantic information. This indicates that good pedestrian detectors need to be extended with a better semantic understanding of images.

Semantic segmentation methods (for example Chen et al., 2014; Long et al., 2015; Badrinarayanan et al., 2015) can classify image pixels into semantic classes such as sky, building, road, vehicle, etc. Semantic segmentation and object detection are two strongly correlated tasks which can be cooperated for better performance in both tasks (Dai and Hoiem, 2012; Trulls et al., 2014; Costea and Nedevschi, 2016). On one side, object detection results can provide valuable information for segmentation. In Yang et al. (2012), object shape information obtained via deformable part-based model detection is combined with color and boundary cues to get improved object segmentation results. On the other side, image segmentation results can facilitate the object detection task. In Yao et al. (2012), the authors formulated the image labeling problem as inference in a conditional random field (Lafferty et al., 2001). They incorporate object reasoning potentials together with segmentation potentials in a unified graphical model for better scene understanding. For the pedestrian detection problem, semantic segmentation can provide valuable complementary information for the localization of pedestrians. Given the background segmentations (such as sky, road, and buildings), some false positives, such as falsely detected pedestrians located on a tree, can be easier eliminated. Meanwhile, the foreground classes such as pedestrian and cyclists can be served as an alternative principle for pedestrian detection. (Costea and Nedevschi, 2016) proposed to use semantic classification cost to facilitate pedestrian detection. They detect pedestrians using sliding windows over filtered feature channels. In their work, traditional “HOG + LUV” features, which is the combination of the Histogram of Oriented Gradient (HOG) (Dalal and Triggs, 2005) feature and three color channel features in the L^*^, u^*^, v^*^ color space are used for image representation. They used decision trees as classifiers for pixel-wise image segmentation. As pixel-wise classification results using decision trees are noisy and inconsistent so that they relied on a conditional random field inference procedure to improve the segmentation results. The performance reported in Costea and Nedevschi (2016) reveals that it is beneficial to improve the detection accuracy by using additional semantic cues.

In our work, we use convolution neural network (CNN) for feature extraction. Compared to hand-craft features, CNN has better capability of feature representation. On the observation that CNN feature maps can be successfully used for the semantic segmentation task (Long et al., 2015; Eigen and Fergus, 2015; Noh et al., 2015), we propose a framework which utilizes a semantic network to improve the performance of Faster-RCNN based pedestrian detector. On the basis of the convolutional feature maps, the semantic network provides higher level semantic feature maps and is integrated with CNN features for classification. A modified version of RPN (Ren et al., 2015) is used to generate a pool of pedestrian hypotheses, meanwhile a semantic network is used to provide additional semantic features. Features from the RPN and the semantic network are integrated and fed into boosted forest (BF) for classification. The semantic network in our proposed deep framework can provide valuable complementary information for pedestrian detection and can, to some extent, be seen as an alternative detection scheme for pedestrians. In such way, successful detection and segmentation require the agreement of both detection and segmentation predictions. In order to ensure good detection accuracy at multiple scales, we jointly use feature maps of multiple resolutions that are extracted from different layers of the two networks. Taking the advantage of BF which imposes no constraint on the dimension of features, the convolutional features and semantic features of different resolutions can be integrated directly. Experimental results show improvements on detection rates using the additional semantic cues.

This paper is organized as follows. In section 2, we provide a brief discussion of related works in terms of pedestrian detection and semantic segmentation. The proposed pedestrian detection framework is introduced in section 3, followed by results discussed in section 4. The conclusions are drawn in section 5.

## 2. Related work

### 2.1. Hand-engineered feature based pedestrian detectors

Histogram of Oriented Gradient (HOG) (Dalal and Triggs, 2005) based detectors using a multi-scale sliding window mechanism have long been the dominant approach for pedestrian detection. While no single hand-craft feature has been shown to outperform HOG, the combinations of HOG with other feature descriptors for different visual cues have resulted in higher accuracy in terms of the achieved low false-positive rate and high true-positive rate. As for example, in Wang et al. (2009), a texture descriptor based on local binary patterns (LBP) (Ojala et al., 2002) was combined with HOG to overcome the problem of partial occlusions. HOG descriptors are used together with LUV color features in the form of image channels features (ICF) in Dollár et al. (2009a). The ICF detector has faster computational speed than HOG as it uses integral images over feature channels. Aggregated channel features (ACF) (Dollár et al., 2014) approximates multi-scale gradients using nearby scales so that it can achieve very fast feature pyramid for real-time multi-scale detection. Checkerboards (Zhang et al., 2015) is a generalization of the ICF, which filters the HOG+LUV feature channels before feeding them into a boosted decision forest.

### 2.2. Region-CNN based pedestrian detection methods

Apart from the dense detection framework using sliding windows scheme, like the HOG detector (Dalal and Triggs, 2005) and its modifications (Wang et al., 2009; Felzenszwalb et al., 2010; Yan et al., 2014; Pedersoli et al., 2015), there is another pipeline of detection methods using “attention” mechanism and is referred to as region-based detection methods (Girshick et al., 2014; Uijlings et al., 2013; Girshick, 2015; Jian et al., 2015, 2017). These methods propose a number of high potential pedestrian candidate regions which is much less than that of sliding window methods. Classifications are performed focusing on the proposal regions so as to be more cost-efficient.

Region-based convolutional neural networks (R-CNN) (Girshick et al., 2014) is a representative region-based detection method using deep neural network (DNN) features. The initial version of the R-CNN detector uses the selective search approach (Uijlings et al., 2013) for region proposal. Despite accurate, R-CNN is too slow for real-time applications even with high-end hardware. Faster R-CNN (Ren et al., 2015) improves R-CNN by replacing selective search (Uijlings et al., 2013) with a built-in network that can directly generate proposals. This sub-network, referred to as region proposal network (RPN), is integrated with Fast R-CNN (Girshick, 2015) to pool candidate object bounding boxes with features extracted using region of interest (RoI) pooling.

Despite Faster R-CNN being particularly successful for object detection, the results for pedestrian detection are not satisfying on pedestrian benchmark (Dollár et al., 2009b). The anchors used in Ren et al. (2015) for generic object detection are of multiple aspect ratios, which may not be suitable for pedestrian detection. Anchors of inappropriate aspect ratios will induce false detections and are harmful for detection accuracy. In Zhang et al. (2016a), the anchors are tailored into a single aspect ratio of a wider range of scales to be suitable for pedestrian detection and this approach achieves promising results on the Caltech dataset.

### 2.3. Semantic image segmentation

Semantic image segmentation, also be referred as semantic image labeling, aims to assign every pixel of an image with an object class label, challengingly combining image segmentation and object recognition in a single process. Before DNN make success on semantic image segmentation, the dominate approaches were Random Forest (RF) based classifiers (Shotton et al., 2008; Yao et al., 2012; Liu and Chan, 2015). The earlier DNN based semantic segmentation approaches (Ciresan et al., 2012) perform classification on image patches. Each pixel was individually classified into a category using a fixed size image patch surrounding this pixel. The reason of using patches was that the deep classification networks usually have full connected layers which require fixed size inputs. In 2015, Fully Convolutional Networks (Long et al., 2015) popularized CNN architectures for dense predictions without any fully connected layers. This allowed segmentation to be performed on a whole image of arbitrary size and also speed up the segmentation process compared to patch-based approaches.

For semantic segmentation problems, pooling layers help in classification networks because they help increase the receptive fields, while on the other hand, pooling decreases the spatial resolution. The “encoder-decoder” architecture was proposed for semantic segmentation approaches (Ronneberger et al., 2015; Badrinarayanan et al., 2015; Noh et al., 2015) to recover the spatial dimension. The encoder gradually reduces the spatial dimension with pooling layers, while a decoder recovers the spatial dimension. SegNet (Badrinarayanan et al., 2015) is such an encoder-decoder deep architecture for pixel-wise semantic labeling. The network consists of a convolutional network (be referred to as the encoder network) and an up-scaling network (be referred to as the decoder network), followed by a classification layer. The feature maps obtained from the upsampling process are sparse. For dense image labeling applications, SegNet converts these sparse feature maps into dense ones using the nearest neighbor approach. As reported, SegNet provides competitive performance using less memory, compared to other state-of-the-arts deep semantic segmentation method (Eigen and Fergus, 2015; Long et al., 2015; Noh et al., 2015).

## 3. Proposed method

**Figure 2:**
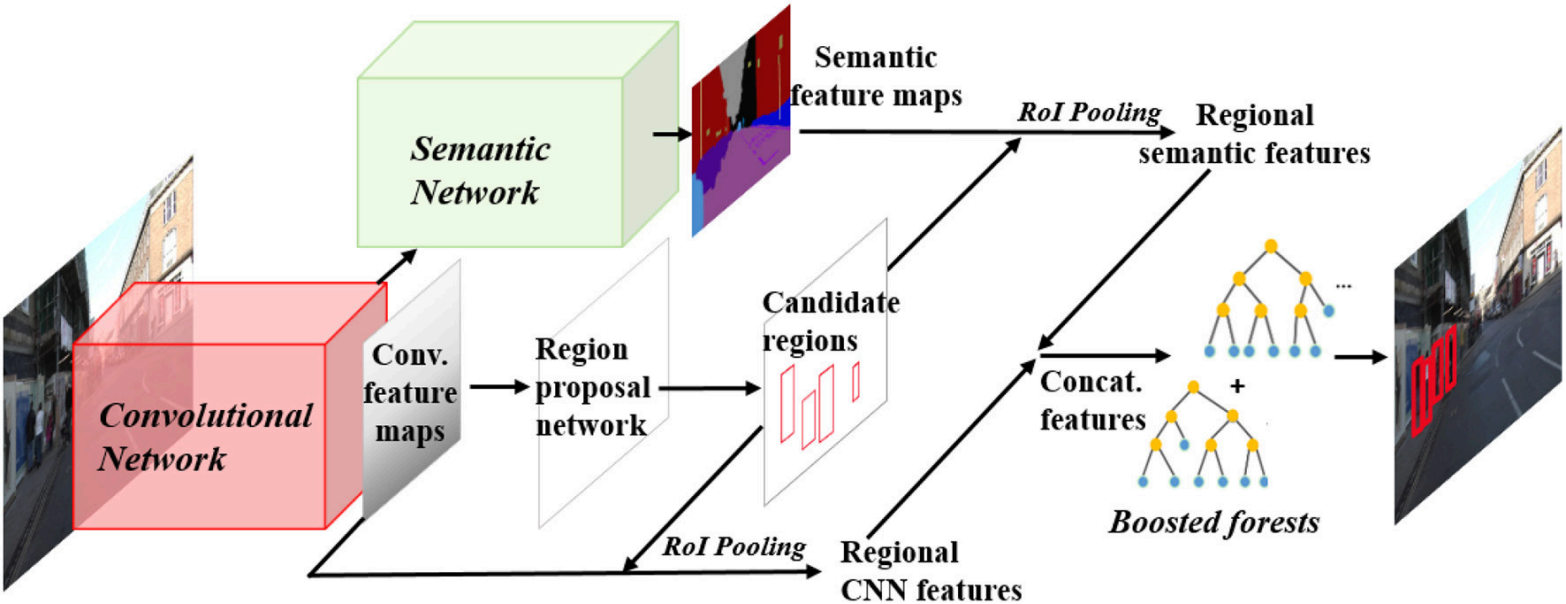
Illustration of the proposed pedestrian detection framework. A semantic network is constructed on the basis of the Faster-RCNN network. For a testing image, the convolutional neural network computes convolutional feature maps, and meanwhile the semantic network computes semantic feature maps to provide complementary semantic features for pedestrian detection. Region Proposal Network (RPN) is used to generate a pool of pedestrian hypotheses. Regional CNN features and regional semantic features for each hypotheses region are pooled via RoI pooling. The integration of CNN features and semantic features are fed into boosted forest for classification.

In this section, we introduce the proposed pedestrian detection framework. As illustrated in Figure 2, a semantic network is built on top of the convolutional network,in parallel with the RPN. For a testing image, the convolutional neural network computes convolutional feature maps, and meanwhile the semantic network computes semantic feature maps to provide complementary semantic features for pedestrian detection. RPN is used to generate a pool of pedestrian hypotheses. Regional CNN features and regional semantic features for each hypotheses region are pooled via RoI pooling. The integration of CNN features and semantic features are fed into boosted forest for classification in a cascade manner for hard negative mining.

### 3.1. Region proposal network for pedestrian candidates proposal

RPN (Ren et al., 2015) is a small network built on top of the *Conv*5_3 (i.e., the third convolutional layer of the fifth convolutional block in the VGG-16 network; Simonyan and Zisserman, 2014). The VGG network architecture was introduced by Simonyan and Zisserman (2014). The VGG16 network consists of five convolutional blocks, two fully-connected layers, and one softmax layer. For the first and second convolutional block, there are two convolutional layers; while for third, forth, and fifth convolutional block there are three convolutional layers. Hence, there are 16 weight layers in total in the VGG16 network. RPN contains two sibling fully connected layers, one for classification and the other for bounding box regression. The cost-function for training RPN contains both classification loss and regression loss. RPN slides over the convolutional feature maps at *Conv*5_3 to perform box regression and classification simultaneously, and outputs a set of bounding boxes associated with confidence scores.

We adopt the anchor mechanism of Faster R-CNN (Ren et al., 2015) to enable simultaneously addressing of multiple scales detection on a single scale testing image. The usage of multi-scale anchors waives the requirement of using feature pyramids to detect multi-scale objects. For pedestrian detection, we follow (Zhang et al., 2016a) and use a single aspect ratio γ (width to height) at 9 scales at each position. γ is set to be 0.41, which is the value used in the Caltech benchmark (Dollár et al., 2009b). For pedestrian aspect ratio normalization.

The RPN network is fine-tuned using the Caltech dataset by fixing the first four convolutional layers. The number of total iterations is 80*k*, where the learning rate of first 60*k* iterations is 0.001, and the learning rate of the last 20*k* iterations is 0.0005. In each mini-batch, there are 128 region of interest (RoIs) from one image. RPN outputs proposals high quality pedestrian proposals. With 100 proposals per image, the PRN can achieve >99% recall at an intersection of union (IoU) of 0.5, and >95% recall at an IoU of 0.7. At test time, RPN outputs top-ranked 100 proposals for classification, while for training RPN outputs the top-ranked 1, 000 proposals.

### 3.2. Semantic network for semantic feature extraction

Semantic segmentation requires dense classification result of the same resolution as the input image. However, CNNs feature maps are of much smaller resolution than the input image due to the effects of pooling layers. Therefore, an upsampling module is required in order to recover spatial resolution of the CNN feature maps. Take VGG16 network for instance, the output feature maps throughout five max-pooling layers of VGG16 are of very low resolution. For example, for an input image of size 480 × 360, the output feature maps throughout five max-pooling layers will shrink by 2^5^ so that the output feature maps are of size 15 × 6. It is required to map the low resolution feature maps into higher resolutions for pixel-wise semantic labeling.

Motivated by the success of SegNet (Badrinarayanan et al., 2015) for semantic segmentation, we make use of the upsampling structure to generate semantic features for our pedestrian detector. SegNet (Badrinarayanan et al., 2015) consists of a convolutional network (be referred to as the encoder network) and an up-scaling network (be referred to as the decoder network), followed by a classification layer. The encoder network is identical to the structure of the VGG-16 network (Simonyan and Zisserman, 2014) of 13 convolutional layers. The decoder network, which is topologically axisymmetric to the encoder network works for upsampling the lower resolution feature maps to higher resolution ones for pixel-wise semantic labeling. At each pooling layer of the encoder network, the pooling indices (i.e., indices of the pixels retained at pooling layers) is memorized during max-pooling to be reused during upsampling. These pooling indices are then passed to the upsampling layers of the decoder network for upsampling.

**Figure 3:**
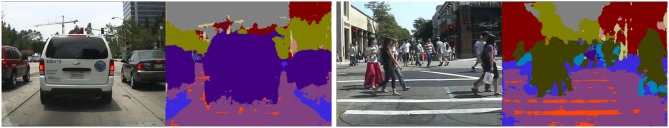
Example semantic segmentation results of SegNet (Badrinarayanan et al., 2015) on the Caltech images.

The semantic network was trained using Caffe-SegNet (Jia et al., 2014; Badrinarayanan et al., 2015). The semantic network is initialized using the VGG-16 network pre-trained on ImageNet (Deng et al., 2009) and fine-tuned on a large database combining a set of urban traffic images (Brostow et al., 2008; Cordts et al., 2015) as in Badrinarayanan et al. (2015). An example of semantic labeling result obtained by SegNet is given in Figure 3. As we can see, semantic segmentation gives reliable background segmentations results for sky (in gray color), road (in light purple), trees (in light green), and buildings (in red). This information can be use to eliminate falsely detected pedestrians located on a tree or in the sky. Meanwhile, SegNet can roughly label the pedestrian regions (in dark green), which can be served as an alternative principle for pedestrian detection.

In SegNet, the high dimensional feature representation at the last upsampling layer is fed to the *argMax* layer to generate the index of class for each pixel. The predicted semantic class corresponds to the class of maximum probability at each pixel position. Such approach has to make a hard decision on pixel-wise classification, so that the rounding errors for semantic classification could hardly be rectified. In our work, we prefer not to make a hard decision at early stage of our pipeline and hence, we do not include an *argMax* layer. Feature maps through the upsampling layers are directly used for semantic feature extraction. The semantic feature maps are used as additional feature channels for the proposed pedestrian detector. A concatenation layer is used after the last upsampling layer in order to integrate the semantic features and the convolutional features.

### 3.3. Multi-channel features for pedestrian detection

Once the RPN has generated the region proposals and confidence scores, we applied RoI pooling to extract fixed length convolutional feature vectors and semantic feature vectors for each candidate regions. While features from deeper CNN layers with higher representative ability are essential for classification, features from shallow CNN layer are of higher-resolution and can be rather useful for detecting small objects like pedestrians. Therefore, we make use of feature maps of multiple resolutions extracted from different layers of the two networks. Unlike in Zhang et al. (2016a) where only convolutional features are extracted and fed into the BF classifier, we cooperate the features from our semantic network.

#### 3.3.1. Multi-resolution convolutional features

**Figure 4:**
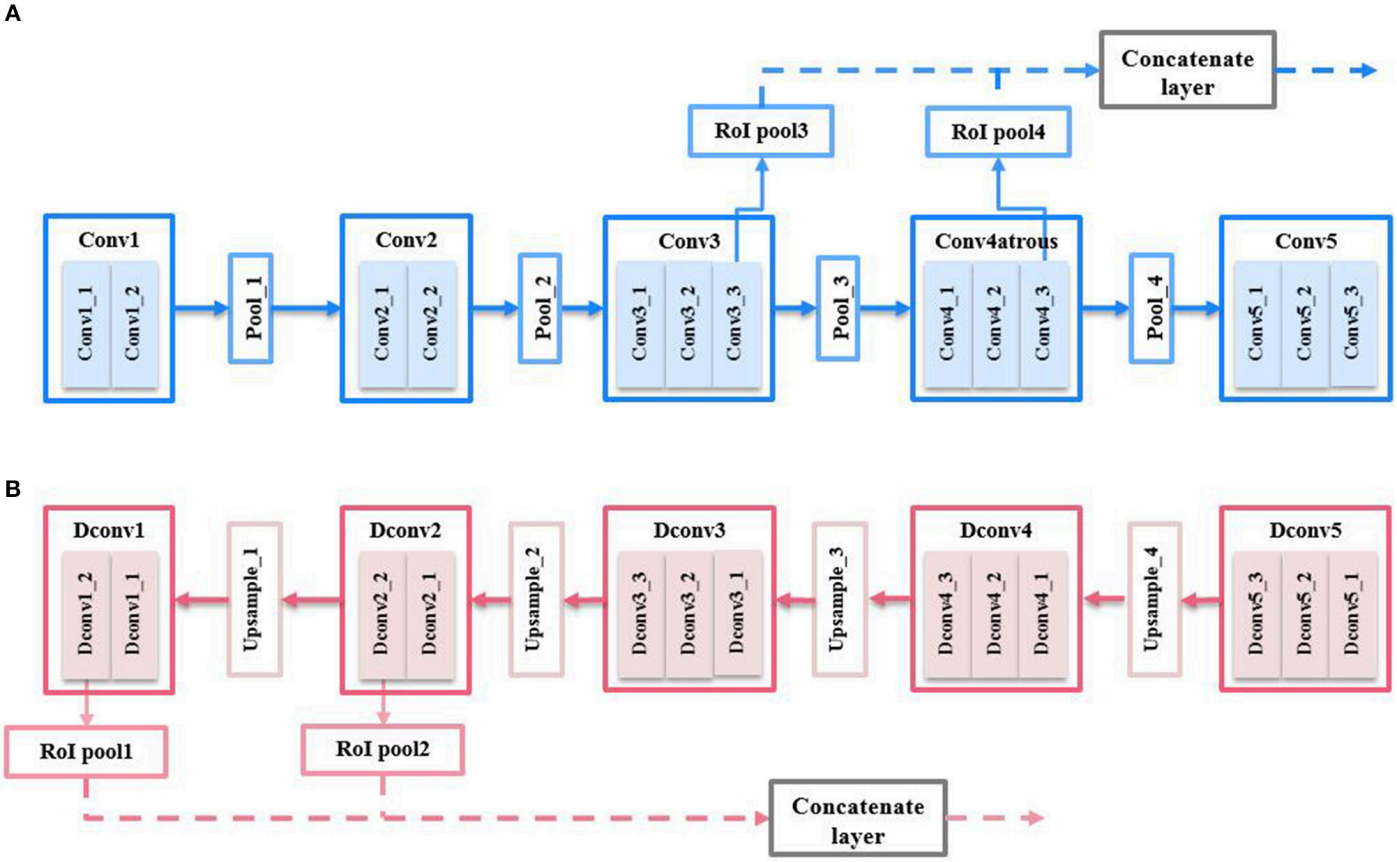
Multi-layers feature extraction for **(A)** CNN feature; **(B)** semantic feature.

The VGG16 network consists of five convolutional blocks, i.e., *Conv*1, *Conv*2, *Conv*3, *Conv*4, and *Conv*5. Only the feature maps of the last convolutional layer, i.e., *Conv*1_2, *Conv*2_2, *Conv*3_3, *Conv*4_3, and *Conv*5_3 will be used for the feature representation of this convolutional block. For simplicity, we will refer to the feature maps output from the last layer of the Xth convolutional block as *ConvX* (*X* = 1, 2…, 5), henceforth. CNN features are extracted from multi-layers of the CNN as illustrated in Figure 4A. In a comprehensive analysis of performance using different layer of CNN features (see section 4.2), we found that the combination of features from *Conv*3 and *Conv*4 gives the best performance.

In addition, we also exploit the “atrous" version of CNN features. The atrous convolution technique is proposed in Chen et al. (2014) which doubles the feature resolution extracted from *Conv*4 to achieve better semantic segmentation performance. An atrous feature map is obtained by dilating the original filter size by a factor of 2 so that the stride of the original feature map can be reduced by 2. Using the *a* trous convolution enables a higher feature resolution while preserving the same feature representation ability. This is crucial for small object detection. Hence, we also perform experiments on the dilated version of *Conv*4 and *Conv*5 features and refer to them as *Conv*4*a* and *Conv*5*a* henceforth. Unlike in Ren et al. (2015) where only *Conv*5 features are used, we combine multi-resolution feature maps from multiple layers of a CNN. Figure 4A illustrates the extraction of multi-layers CNN features. As will be given in section 4.2, the combination of features from *Conv*3 and *Conv*4 gives the best performance among the convolutional features.

#### 3.3.2. Multi-resolution semantic features

Semantic features are extracted from multi-layers of the semantic upsampling network as illustrated in Figure 4B. The networks structure is illustrated briefly without showing ReLU layers and batch normalization layers for simplicity. The upsampling layers of the semantic network are denoted as *DconvX* (*X* = 1, 2…, 5), which represent the *X*th upsampling block of the semantic network. We extracted semantic features from both *Dconv*1 and *Dconv*2 which provide the best performance (as it will be shown in section 4.2).

We use RoI pooling (Girshick, 2015) to extract fix-length semantic feature vectors for candidate regions of arbitrary size. For each feature map channel, RoI pooling works by maximizing the values in each sub-window into the corresponding output grid cell. A region proposal of arbitrary size is partitioned into 10 × 5 sub-windows along the vertical and horizontal directions. The semantic features are integrated with convolutional feature though a concatenation layer and are fed into the BF classifier for detection.

### 3.4. Boosted forest for integrated multi-channel features

Boosted Forest (BF) is an ensemble learning method which can achieve fast and accurate classification. Owing to high accuracy and low computation cost, decision forests have been widely used in computer vision tasks such as object recognition (Wohlhart et al., 2012; Gall and Lempitsky, 2013), and super-resolution (Huang et al., 2015; Huang and Siu, 2017; Jun-Jie Huang and Stathaki, 2017). In this section, an integration of features from the convolutional network and the semantic network are trained using BF to create a strong classifier. The flexibility of BF for various dimensions of features is convenient for us to combine the lower-resolution semantic features with higher-resolution convolutional features. We adopt the RealBoost (Schapire and Singer, 1999) algorithm with bootstrapping which is effective for mining hard detection samples. As the output of RPN contains both the bounding box position and the confidence score, these scores are used as preliminary scores for the boosted classier.

To train the decision forests, we use seven bootstrapping rounds, each stage has {64, 128, 256, 512, 1, 024, 1, 536, 2, 048} trees. Initially, the training set consists of all positive examples and 20, 000 negative samples. In the first training stage, negative training samples are randomly generated, avoiding the regions containing pedestrians. For the other six stages, hard negative samples are selected using the detector trained from the previous stage. The number of hard negative samples to be added after each bootstrapping round are limited to 40, 000. The BF classifier obtained at the final stage is used for testing. We also built a basic version of the detector, which allows us to exploit many more different settings in a short training time. The basic detectors has five training stages. Each stage has {32, 64, 128, 256, 512} weak classifiers, respectively. At the first stage, 10, 000 negative samples are randomly sampled and, the number of hard negative samples to be added at each bootstrapping pass is limited to 1,000.

## 4. Experiments

### 4.1. Datasets and implementations

The proposed pedestrian detector is trained on the Caltech benchmark (Dollár et al., 2009b). With improved annotation (Zhang et al., 2016b). The Caltech pedestrian dataset consists of approximately 10 hours of 640 × 480 video taken from a vehicle driving through urban areas. It contains about 250, 000 frames of over about 2, 300 unique pedestrians. We generated the Caltech3x dataset by extracting the image frames every 10th frame from the Caltech videos. For the basic detectors, the Caltech training dataset with 4, 250 frames extracted every 30 frames from the videos is used. There are 4, 024 frames in the Caltech testing set. The performance is evaluated under “reasonable” evaluation setting where only pedestrians above 50 pixels in height without serious occlusions are counted. We measure the log average miss rate ranging from 10^−2^ to 10^0^ (i.e., *MR*_−2_) and 10^−4^ to 10^0^ (i.e., *MR*_−4_) false positive per image (FPPI) for evaluation (Dollár et al., 2009b).

The implementation is based on the public available code for Faster-RCNN (Zhang et al., 2016a; Jia et al., 2014) and object detection toolbox (Dollar, 2012). All experiments were run on a machine with a single GPU TITAN X and a CPU Intel Core i7 3.4GHz.

### 4.2. Results and discussions

#### 4.2.1. Results of multi-resolution semantic features

First, we conduct experiments using different convolutional layers to find the most suitable CNN features. Feature representation from a single convolutional layer, i.e., *Conv*1, …, *Conv*3, *Conv*4/*Conv*4*a* and *Conv*5/*Conv*5*a*, is used for training and the results are compared in Table 1. These experiments are tested using same set of RPN proposal. We measured the averaged log miss rates of the detectors over the FPPI range of [10^−4^, 100] (*MR*_−4_). As we can see, the best two performances are achieved by using *Conv*3 and *Conv*4*a* features. The best two performances are achieved by using *Conv*3 and *Conv*4*a* features (bold in Table 1). Features from a deeper CNN layer have stronger representation ability but lower resolution, whereas features from a shallower layer is of higher resolution but weaker representation ability. For the pedestrians detection task, features from shallower layers, i.e., *Conv*1 and *Conv*2, have too weak feature representation capability. On the other hand, features from the deepest layer, i.e., *Conv*5, has too low resolution for detecting the small pedestrian.

**Table 1:** Comparison of performance (in terms of *MR*_−4_%, the lower the better the top two permanence is bold) on the Caltech validation set using features from different layer(s) of convolutional network.

***Conv*1**	***Conv*2**	***Conv*3**	***Conv*4**	***Conv*5**	***Conv*4*a***	***Conv*5*a***
36.39	30.39	**20.32**	20.5	31.47	**15.46**	19.16

On the basis of the results using CNN features in Table 1, we combined the two layers of features that lead to the best two results (bold in Table 1). The performance using the concatenation of CNN features from the *Conv*3 layer and the *Conv*4*a* layer, denoted as *Conv*3 + *Conv*4*a* in the first row of Table 2, achieves 13.82 in terms of *MR*_−4_%. Then we evaluate the results of using additional semantic features from different upsampling layer of the semantic network. These experiments are tested using same set of RPN proposal with same parameter setting except the semantic features. The results are given in Table 2. As we can see, by using the semantic features from *Dconv*1 and *Dconv*2, we achieve the best result of 12.65% in terms of *MR*_−2_ (as bold in Table 2).

**Table 2:** Comparison of performance (in terms of *MR*_−4_% the lower the better, the best permanence is bold) on the Caltech validation set using features from different layer(s) of semantic networks.

**ROI features**	***MR*%**
*Conv*3 + *Conv*4*a*	13.82
+*Dconv*1	13.65
+*Dconv*2	13.59
+*Dconv*3	14.55
+*Dconv*4	15.61
+*Dconv*1 + *Dconv*2	**12.65**
+*Dconv*2 + *Dconv*3	13.73

For the reasonable evaluation setup, we get an overall improvement of 1.07 owing to the usage of semantic features. When we look at the fine-grained improvements for different scale ranges, we find that semantic channels make larger improvement for small pedestrians of [50, 80) pixels in height, which is generally a harder case for the pedestrian detection (see Table 3).

**Table 3:** Improvements of semantic network in different scale of pedestrian, testing on the Caltech validation dataset.

**Pedestrian scales**	**Baseline**	**+ Semantic**	**MR%**
(80, Inf]	7.49	6.04	+ 1.45
(50, 80]	17.55	15.09	+ 2.46

#### 4.2.2. Comparison with state-of-the-art pedestrian detection methods

**Figure 5:**
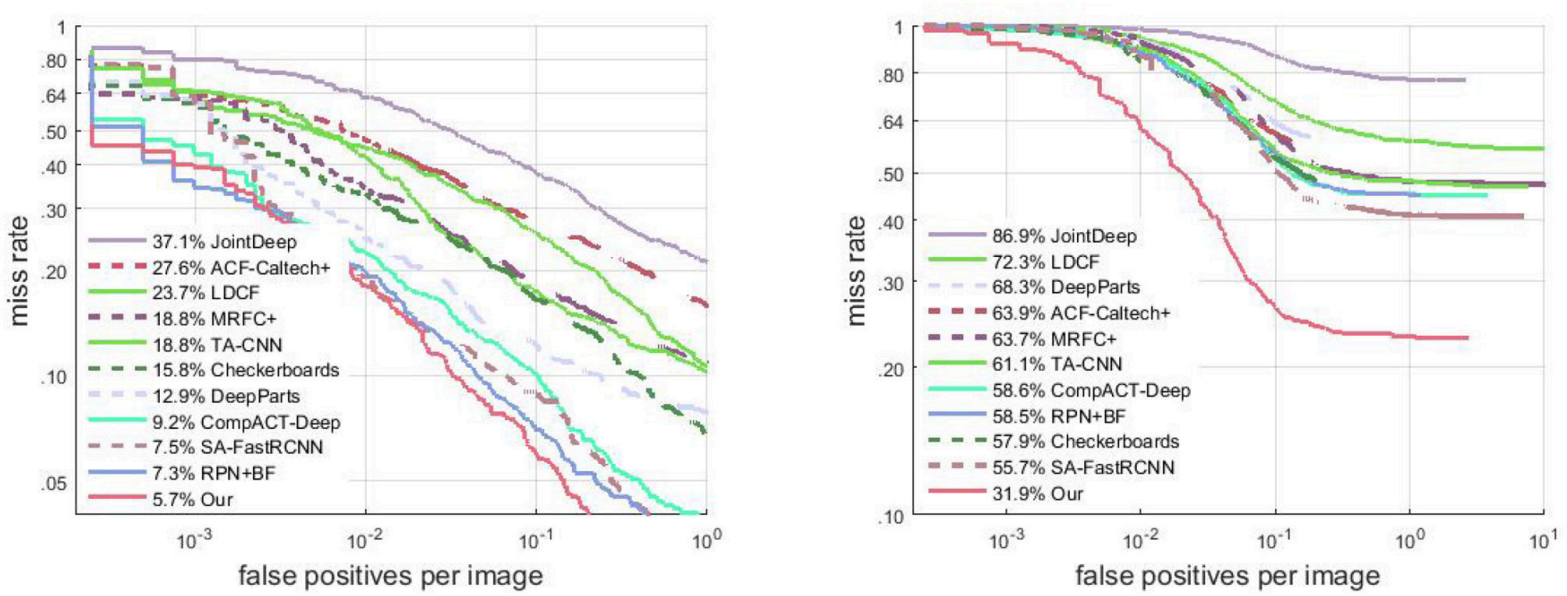
Comparison of results (*MR*_−2_) on the Caltech testing set evaluated using IoU of 0.5 **(Left)** and of 0.75 **(Right)**, respectively.

We compare the proposed pedestrian detector with the state-of-the-art pedestrian detection methods, including ACF (Dollár et al., 2014), LDCF (Nam et al., 2014), Checkerboards (Zhang et al., 2015), MRFC+ (Costea and Nedevschi, 2016), CompACT-Deep (Cai et al., 2015), DeepParts Tian et al. (2015), SA-FastRCNN (Li et al., 2015), and RPN+BF (Zhang et al., 2016a) in Figure 5. Among the comparison methods, ACF, LDCF, Checkerboards are all methods using decision trees for classification; TA-CNN, JDN, CompACT-Deep, DeepParts, SA-FastRCNN and RPN+BF are deep learning based methods; MRFC+ is a recent pedestrian detection works which use semantic features to improve HOG+LUV feature based pedestrian detection.

Detection results on the proposed pedestrian detector on trained using Caltech3x set is given in Figure 5. Under the evaluation setting of intersection of union (IoU) of 0.5, our method has an *MR*_−2_ of 5.7%, which is a improvement of 0.8% beyond the state-of-the-art method (Zhang et al., 2016a). This comparison demonstrate the beneficial of the semantic net which provides additional detection cues. Under a stricter evaluation condition of IoU = 0.75 (see Figure 5, right), our proposed detector outperform other pedestrian detection methods with a large margin. This indicates that our proposed method not only achieves lower miss rate, but also obtains detection with more precise position.

**Figure 6:**
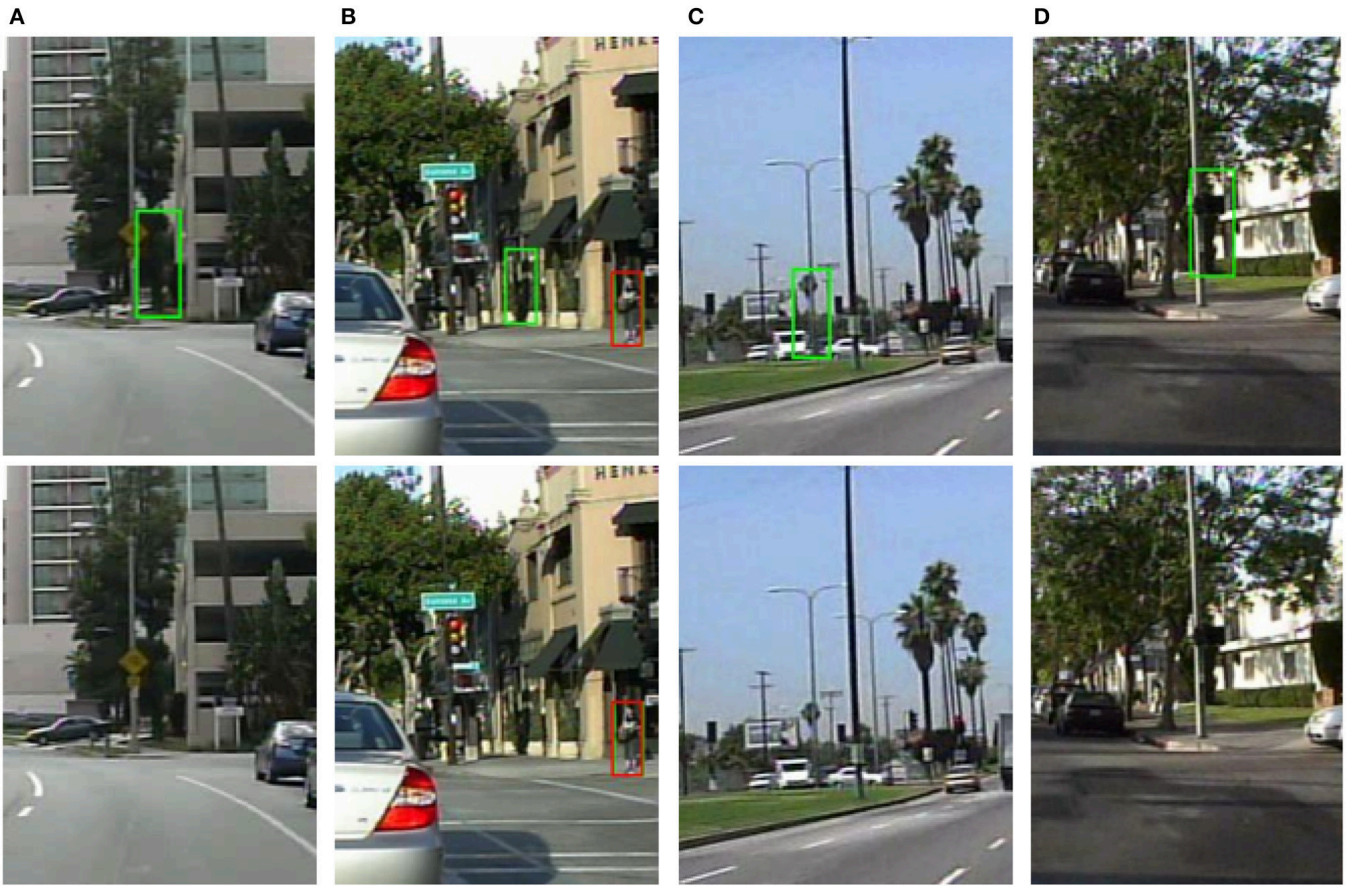
Visualization of example false positives (upper row) removed by the semantic features. The red and the greed bounding box indicates the ground truth pedestrian and detected pedestrian, respectively.

Figure 6 shows some detection results examples where hard negatives are removed using the additional multi-channel features. We can see that there are some ambiguous pedestrian hypotheses, such as trees, which are difficult to be discriminated using CNN feature only. These false positives have been successfully removed by the proposed detector using the semantic features.

## 5. Conclusions

In this paper, we proposed a pedestrian detector which makes use of semantic image segmentation information. Basis on the Faster-RCNN framework, we have unified the detector with a semantic segmentation network. Semantic features extracted from the semantic network are used jointly with convolutional features for improved pedestrian detection. Some ambiguous pedestrian hypotheses that may be difficult to classify from the convolutional feature maps can be discriminated with the help of semantic information on and around each hypothesis. Experiments on the Caltech dataset indicate that the proposed detector make improvement on the baseline detector by enforcing the consistency between the detection network and the semantic network. The proposed solution provides a more powerful pedestrian detector achieving competitive results on pedestrian detection benchmarks at 0.21 s per frame on single TITAN-X Pascal GPU. In the future, we can explore the proposed method with more advanced deep neural network such as the residual network (He et al., 2016) for better segmentation and detection performance.

## Author contributions

All authors listed have made a substantial, direct and intellectual contribution to the work, and approved it for publication.

### Conflict of interest statement

The authors declare that the research was conducted in the absence of any commercial or financial relationships that could be construed as a potential conflict of interest.
